# Key pathways in prostate cancer with SPOP mutation identified by bioinformatic analysis

**DOI:** 10.1515/med-2020-0237

**Published:** 2020-10-09

**Authors:** Guanxiong Ding, Jianliang Sun, Lianhua Jiang, Peng Gao, Qidong Zhou, Jianqing Wang, Shijun Tong

**Affiliations:** Department of Urology, Huashan Hospital, Fudan University, 12 Central Urumqi Rd, Shanghai 200040, People’s Republic of China; Department of Urology, The Affiliated Suzhou Hospital of Nanjing Medical University, 26 Daoqian Rd, Suzhou, Jiangsu 215000, People’s Republic of China

**Keywords:** prostate cancer, bioinformatic analysis, SPOP mutation, TCGA, RNA sequencing

## Abstract

Prostate cancer (PCa) is a leading adult malignant tumor. Recent research has shown that speckle-type BTB/POZ protein (SPOP) mutant is the top frequently mutated gene in PCa, which makes it an important biomarker. In this paper, we aimed at identifying critical genes and pathways related to SPOP mutation in PCa. Recent The Cancer Genome Atlas data showed that 12% of patients with PCa were SPOP mutant. There were 1,570 differentially expressed genes, and online enrichment analysis showed that these genes were mainly enriched in metabolism, pathways in cancer and reactive oxygen species. INS, GNG13, IL6, HTR5A, SAA1, PPY, CXCR5, CXCL13, CD19 and CCL20 were identified as hub genes. The lower SPOP expression level was associated with poor prognosis. In all, our findings showed that various pathways and genes could play critical roles in SPOP mutation in PCa, providing potential options for individualized treatment.

## 1 Introduction

Prostate cancer (PCa) is the second most common malignant tumor in men worldwide after lung cancer. A total of 12,76,106 new cases were reported in 2018, of which 3,58,989 resulted in death (3.8% of all cancer deaths in men) [1]. The incidence and mortality of PCa worldwide are related to the increase in age, and the average age at diagnosis is 66 years. It is worth noting that compared with white men, African Americans have a higher morbidity rate, with 158.3 new cases diagnosed per 1,00,000 men, and the mortality rate is about twice that of white men [2]. The reason for this difference may be due to the difference in social, environmental and genetic factors. It is estimated that the global incidence of PCa will increase by 10,17,712 cases in 2040 (a total change of 79.7%): the highest incidence of PCa will be in Africa (+120.6%), followed by Latin America and the Caribbean (+101.1%) and Asia (100.9%) [3]. Therefore, the prevention and treatment of PCa is of great importance.

The mechanism of the initiation and progression of PCa is still not very clear, and therefore its early diagnosis and personalized treatment are not possible. In spite of the changes in critical genes and pathways, numerous studies have indicated that copy number alterations, somatic mutations and oncogenic structural DNA rearrangements (chromosomal abnormalities) might play important roles in primary PCa, metastatic PCa and metastatic castration-resistant prostate cancer (mCRPC) [4,5,6,7]. Therefore, to further explore the functions of such mutations in PCa, development in sequencing research helps in understanding the disease program, which identifies some molecular markers applicable in clinical practice.

With continuous development in sequencing research, many key gene mutations have been found in PCa, which has promoted the identification of the disease and the search for biomarkers. Among these key genes, speckle-type BTB/POZ protein (SPOP) mutation is a top frequently mutated gene in PCa, which makes it a promising biomarker for PCa personalized treatment options [5,7]. SPOP encodes a protein that modulates the transcriptional repression activities of death-associated protein 6 (DAXX), which plays major roles in physiological and pathological programs in our body by interacting with histone-associated proteins [8]. SPOP mutation has been known to play important roles in the progress and treatment of PCa [9,10,11]. Recent research confirmed that patients with mCRPCs with SPOP mutations often have a deletion of CHD1, which is highly sensitive to abiraterone treatment [12]. Therefore, whether SPOP mutation can be used as a biomarker of disease to contribute to individualized treatment is a question worthy of study.

In this study, we analyzed the gene expression data set of PCa in The Cancer Genome Atlas (TCGA) to determine the role of SPOP mutation in disease progression. We also identified critical pathways and hub genes associated with SPOP mutation to determine the potential mechanisms and therapeutic targets. Our findings may provide novel individualized treatment options for PCa.

## 2 Materials and methods

### 2.1 Identification of differentially expressed genes (DEGs)

We use edgeR to examine the RNA-sequencing data to explore DEGs between patients with PCa with SPOP mutation and wild-type group [13,14]. The criteria are as follows: *P*-value < 0.05 and |fold change (FC)| ≥ 2.

### 2.2 RNA-Seq data

A PCa RNA-Seq data set (project: TCGA-PRAD; study accession: phs000178) was obtained directly from the TCGA database. The corresponding clinical information was downloaded from the cBioPortal website [15].

### 2.3 Functional annotation enrichment analysis

Enrichment analysis, such as KEGG analysis and GO analysis, was carried out by the DAVID: database for annotation, visualization and integrated discovery [16].

### 2.4 Gene set enrichment analysis (GSEA)

Differences in the expression levels and pathways of biological function annotation genes between SPOP-mutant and wild-type patients were determined using GSEA software. *P*-value < 0.05 with a false discovery rate (FDR) *q*-val < 0.25 was considered statistically significant. The number of permutations was set to be ten.

### 2.5 Protein–protein interaction (PPI) network and module analysis

We used the search tool for the retrieval of interacting genes (STRING) database, which provides critical PPI evaluation and integration to handle protein interaction analysis [17]. We uploaded all DEGs to STRING to evaluate the interaction. An experimentally valid interaction with an interaction score greater than 0.4 was chosen to be ideal. We did module screening in Cytoscape using molecular complex detection (MCODE) (score > 3 and nodes > 4) [18].

## 3 Statistical analysis

We used an unpaired *t*-test to compare SPOP mRNA expression between SPOP-mutant and wild-type PCa tissues. Clinical outcomes between different gene states were calculated using the Kaplan–Meier method of logrank test in GraphPad. We adjusted FDR in GSEA and edgeR for multiple tests to control FDR via the Benjamini–Hochberg program [19,20]. All statistical analyses were performed using GraphPad and R 3.3.0. *P* < 0.05 was considered statistically significant.

## 4 Results

### 4.1 Data information

**Figure 1:**
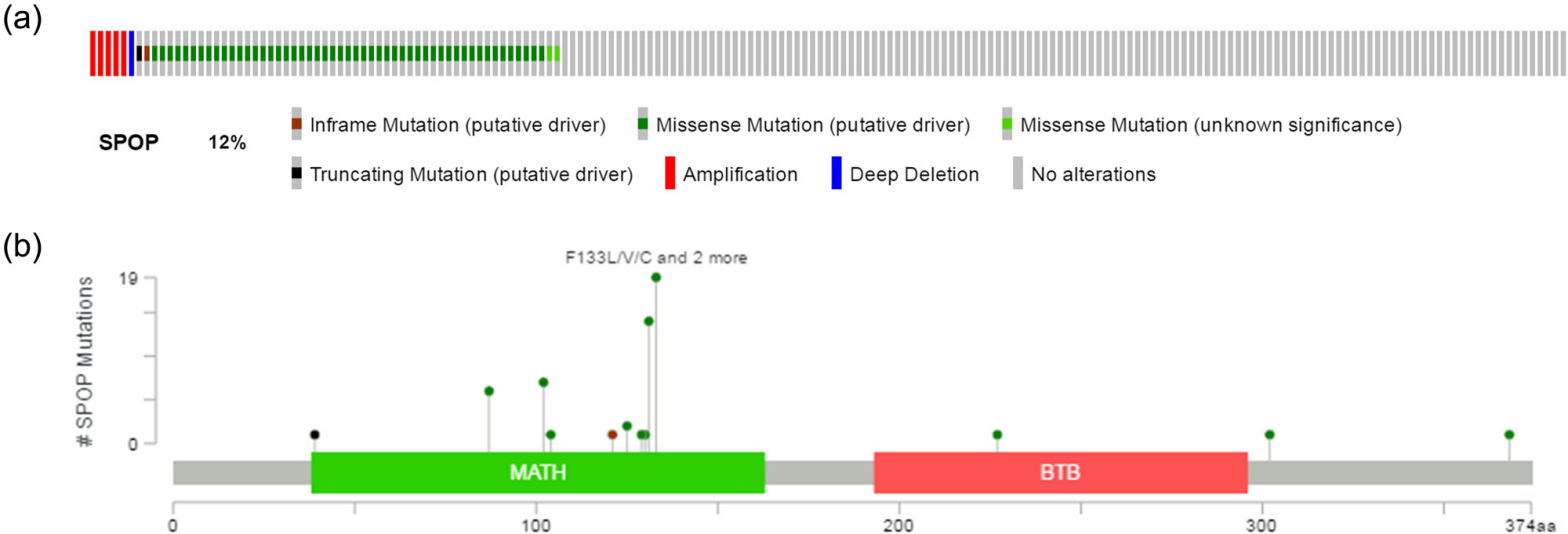
(a) Mutation frequency and (b) types of SPOP in PCa reproduced from TCGA database.

We downloaded the gene expression matrix of 499 patients with PCa directly from TCGA. Sixty-one patients (12%) were identified with SPOP mutation (Figure 1a). Mutation types include truncation, amplification, deep deletion and missense mutation across the entire gene. Of these, missense mutation is the most common type of mutation (Figure 1b). Mutation data were directly obtained from the cBioPortal.

### 4.2 GSEA

**Figure 2:**
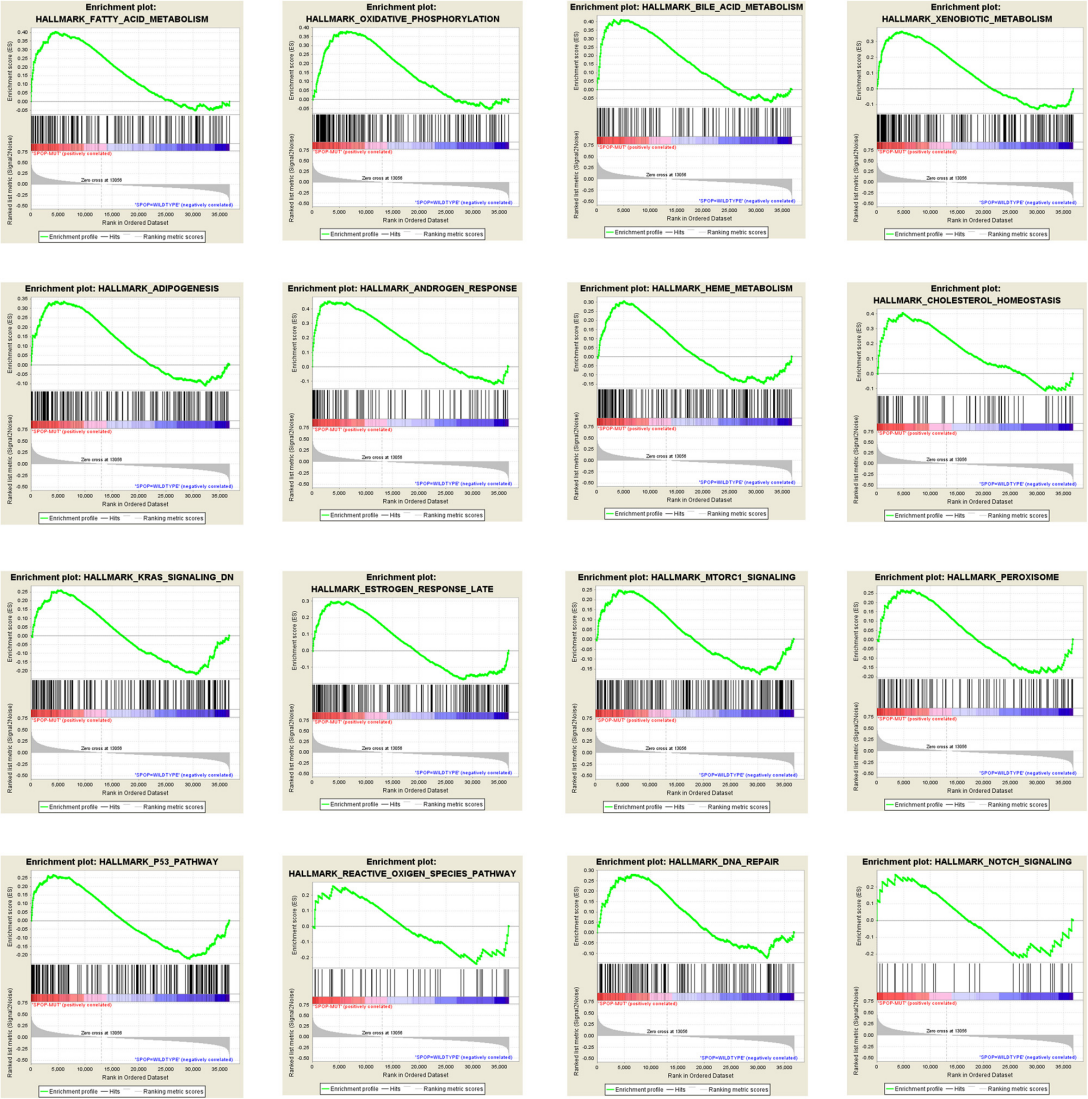
GSEA results of SPOP mutation in patients with PCa.

To further study the function of SPOP mutation on the disease program of patients with PCa, we investigated functional gene set enrichment by GSEA. Figure 2 shows that enrichment is mainly associated with biological processes including fatty acid metabolism, oxidative phosphorylation, bile acid metabolism, xenobiotic metabolism, adipogenesis, androgen response, heme metabolism, cholesterol homeostasis, KRAS signaling, estrogen response, mTORC1 signaling, p53 pathway, reactive oxygen species (ROS) pathway, DNA repair, NOTCH signaling and E2F targets. The result indicates that SPOP mutation might influence disease progression and prognosis by affecting androgen signaling, ROS, DNA repair and metabolism.

### 4.3 Identification of DEGs

**Figure 3:**
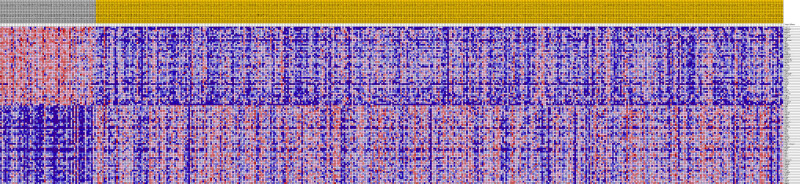
Heat map of the top 100 DEGs. Red: up; purple: down.

**Figure 4:**
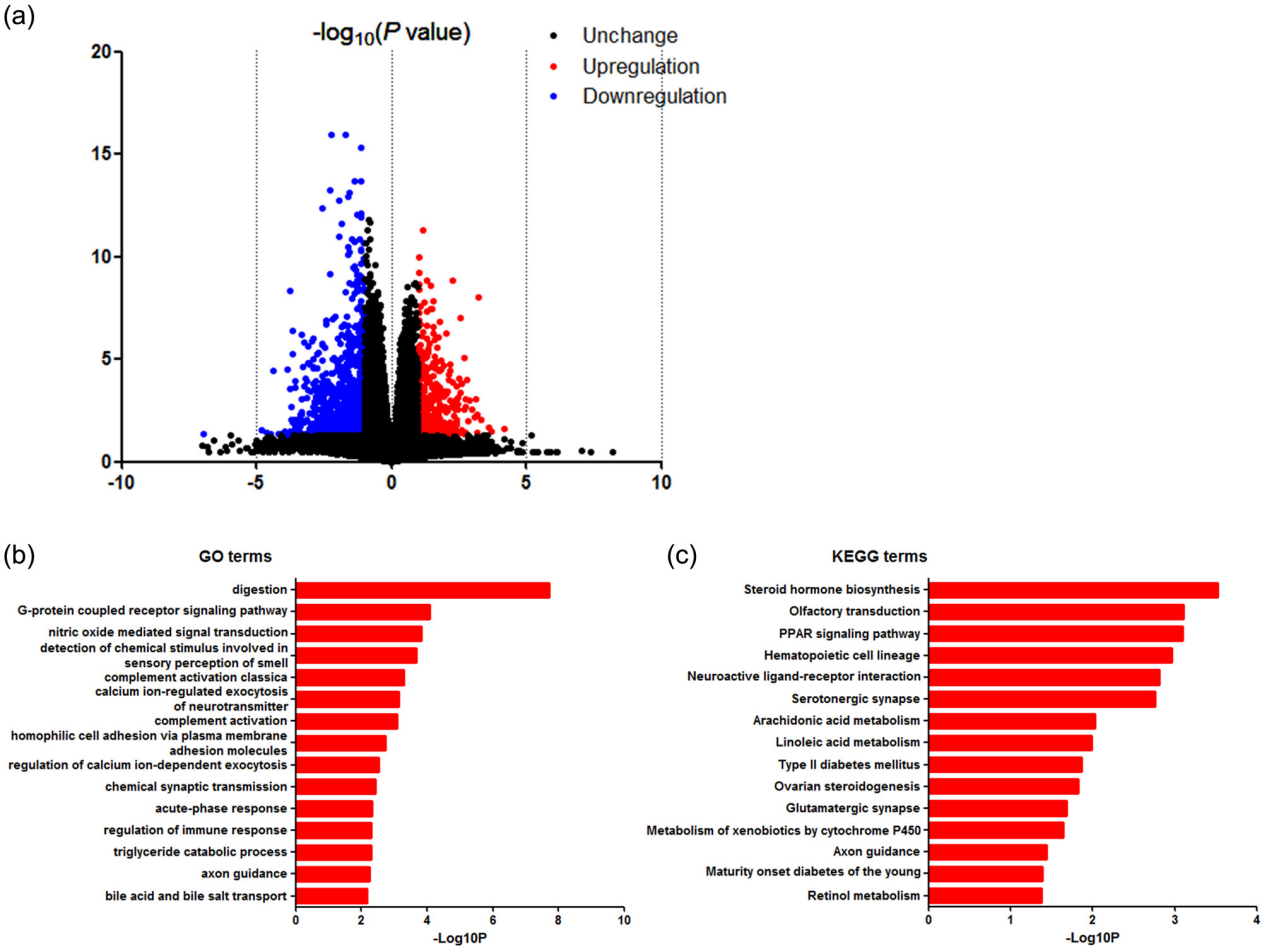
DAVID enrichment results of DEGs. (a) Volcano plot for DEGs. (b) The GO enrichment results of DEGs. (c) The KEGG pathway analysis of DEGs.

The RNA expression data given earlier were used for DEG screening. Based on the *in silico* analysis, 1,570 genes were recognized as DEG: 355 were upregulated and 1,215 were downregulated. Figure 3 shows the heat map of DEG expression for the top 100 genes, and Figure 4a shows the volcano map for DEGs.

### 4.4 Enrichment analysis of DEGs

We used all 1,570 DEGs for the online KEGG pathway and GO analysis. The results of the GO analysis (Figure 4b) suggested significant enrichment in digestion, G-protein-coupled receptor signaling pathway, nitric oxide-mediated signal transduction, detection of chemical stimulus involved in sensory perception of smell, complement activation, classical pathway, calcium ion-regulated exocytosis of neurotransmitter, homophilic cell adhesion via plasma membrane adhesion molecules, regulation of calcium ion-dependent exocytosis, chemical synaptic transmission, acute-phase response, regulation of immune response, triglyceride catabolic process and axon guidance.

In addition, DEGs were significantly enriched in steroid hormone biosynthesis, olfactory transduction, PPAR signaling pathway, hematopoietic cell lineage, neuroactive ligand–receptor interaction, serotonergic synapse, arachidonic acid metabolism, linoleic acid metabolism, type 2 diabetes mellitus, ovarian steroidogenesis, glutamatergic synapse, metabolism of xenobiotics by cytochrome P450 and axon guidance in the KEGG pathway analysis (Figure 4c).

### 4.5 Module screening

**Figure 5:**
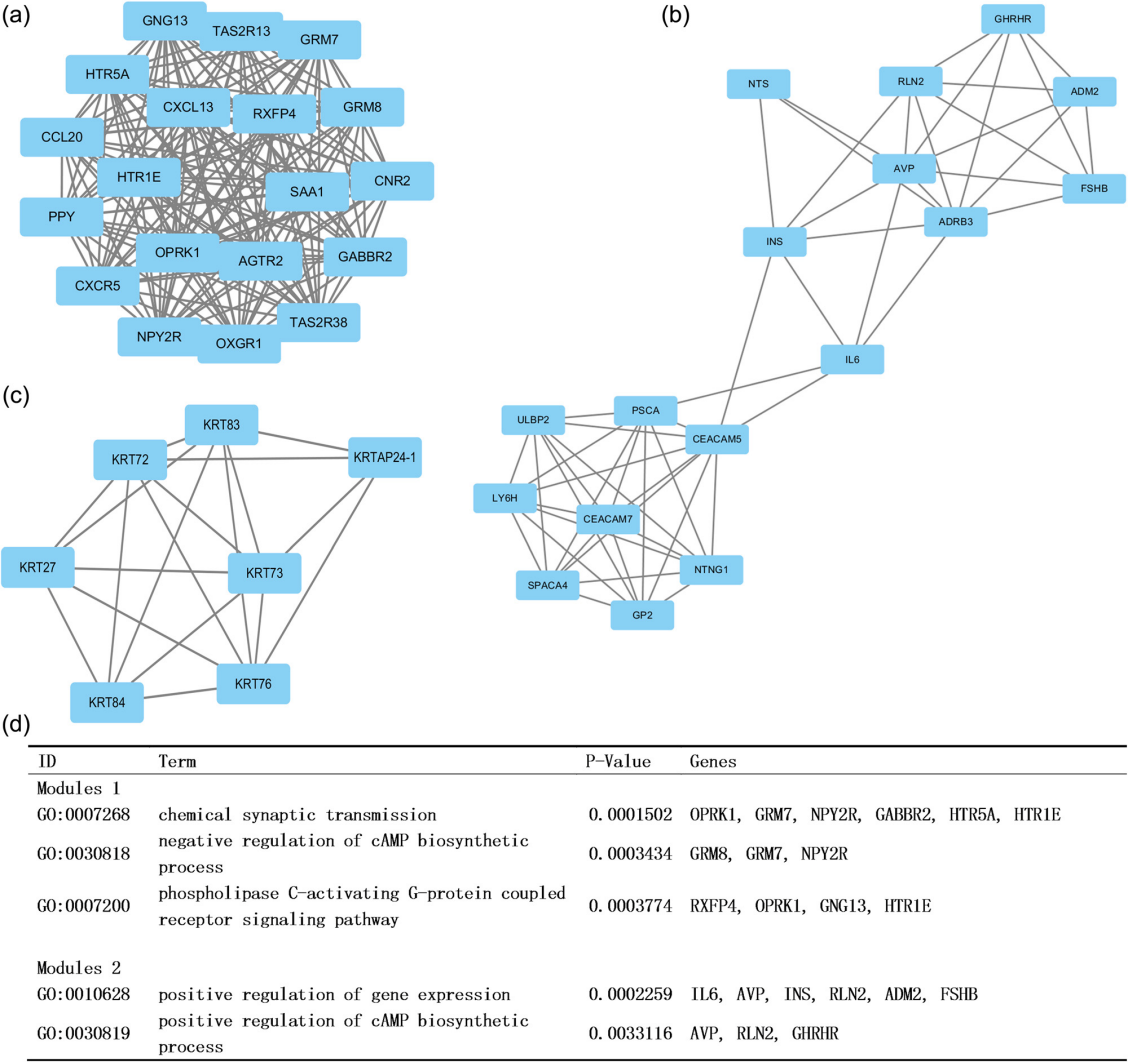
First three modules of protein interaction in the PPI network. (a–c) Results of protein interaction analysis of modules 1–3; (d) GO analyses of the top two modules.

We studied interactions and hub genes by screening in the STRING database. The hub genes were identified to be the top ten genes ranked by degree. The results showed that the hub genes were INS, GNG13, IL6, HTR5A, SAA1, PPY, CXCR5, CXCL13, CD19 and CCL20. INS was the first hub gene with the highest degree of 73. MCODE helped us to analyze the gene modules in the PPI network. Then, we performed enrichment analysis based on the first three important modules (Figure 5). GO analysis indicated that the genes in module 1 were mainly related to chemical synaptic transmission, negative regulation of cAMP biosynthetic process and phospholipase C-activating G-protein-coupled receptor signaling pathway, while positive regulation of gene expression and positive regulation of cAMP biosynthetic process were enriched in module 2.

### 4.6 Clinical effect of SPOP mutation on PCa progression

**Figure 6:**
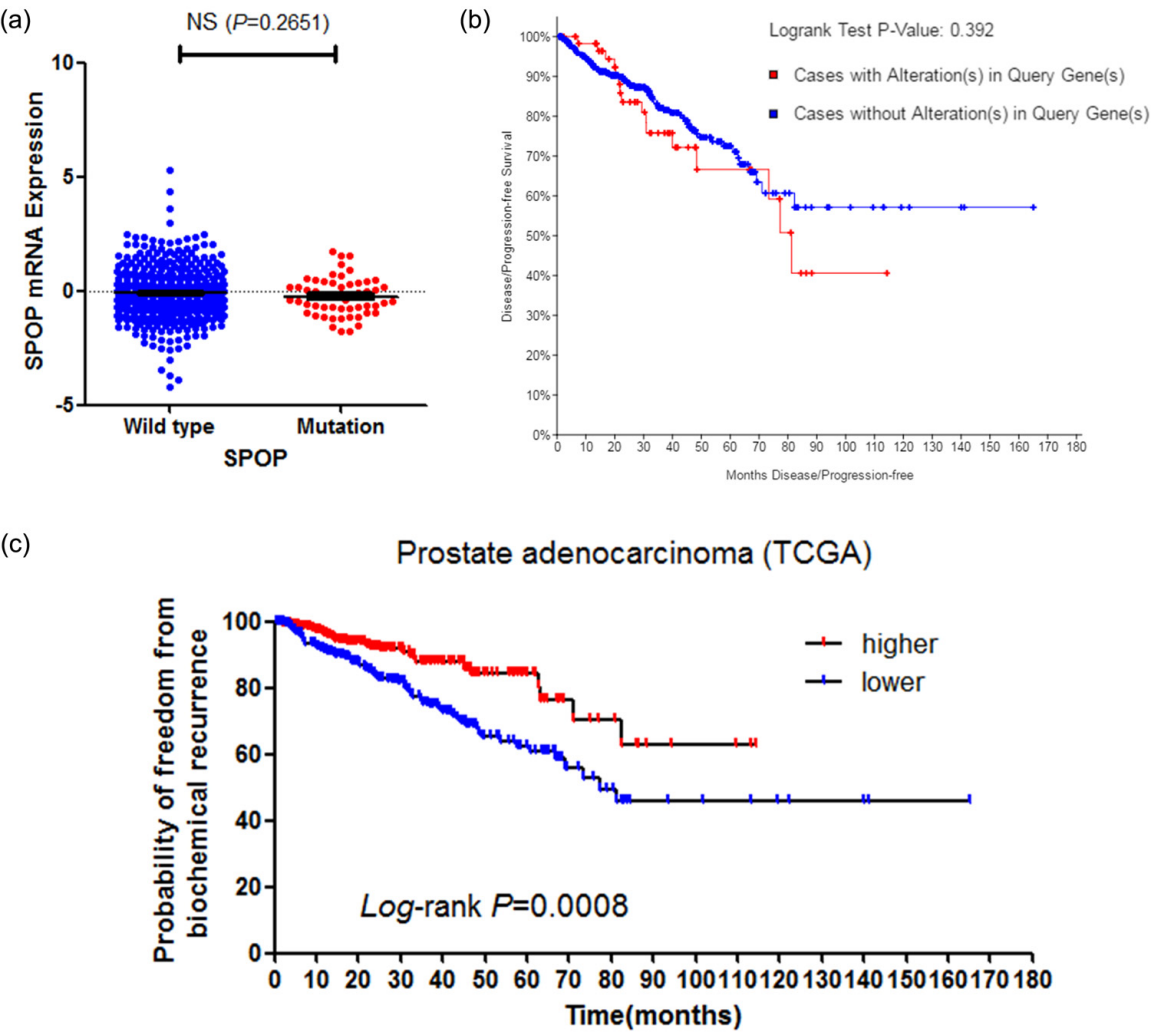
SPOP mRNA level, but not mutations of SPOP, associated with PCa prognosis. (a) SPOP mutation and mRNA expression. (b) Curves of patients with PCa stratified by SPOP mutation. (c) Lower SPOP mRNA levels associated with worse prognosis in PCa.

We investigated the effect of SPOP mutation on PCa progression. We first determined the level of SPOP mRNA expression. The results showed no significant difference in SPOP mRNA level between SPOP-mutant and wild-type patients’ tumor tissues (Figure 6a). However, SPOP mutation was not found to correlate with PCa recurrence (Figure 6b). Survival curves of patients with PCa stratified by SPOP mRNA levels indicated that patients with lower SPOP mRNA expression have a poorer prognosis (Figure 6c).

## 5 Discussion

PCa is one of the most common male malignant tumors, and treating advanced PCa [21] is still a challenge. Radical prostatectomy is a common treatment for clinically localized PCa [22]. The treatment option for patients with PCa who cannot undergo surgery is toreduce the levels of androgen [23,24]. However, almost all patients with PCa eventually develop CRPC after treatment [25,26,27]. The large-scale and multidimensional analyses of human PCa genomics provide comprehensive profiles of the cancer genomic alterations, which enable the development of therapies that target these changes as well as prognosis that identifies patients who may benefit from these therapies [7,28].

We found that SPOP mutation may affect disease progression and prognosis by influencing androgen signaling. KEGG, GO and GSEA analyses all indicated that SPOP mutation in PCa influences metabolism progression, including steroid hormone biosynthesis, fatty acid metabolism, adipogenesis, androgen response and cholesterol homeostasis. All these processes are essential for the metabolism of androgens in the body, which can affect not only the synthesis of androgen but also the biology of androgen and androgen receptor *in vivo*. Our results suggest that the application of anti-androgen therapy may have a gap between patients with SPOP mutation and wild-type patients.

We also found that SPOP mutation might influence the choice of treatment in PCa. Radiation is a common viable treatment option for localized PCa. As an alternative to surgery, it provides high biochemical control, low risk of complications, minimal duration of treatment and outpatient treatment opportunity [29]. Nonetheless, using the current regimen of high-dose conformal radiation, treatment failure occurs in 45% patients with the locally confined disease, which could be caused by increased basal ROS [30]. It might reduce damage sensitivity by inhibiting PTEN expression, enhancing the activity of the PI3K/AKT pathway and reducing ROS production [31]. Our findings showed that patients with SPOP mutation might induce the ROS activity, which results in the failure of radiation treatment in PCa. Other treatments may be more appropriate for such patients.

As for the clinical affairs, our results indicated that SPOP mutation does not correlate with the expression of the SPOP mRNA level in PCa tissue. Survival analysis showed that SPOP mutation does not associate with the poorer or better prognosis of patients with PCa. However, mRNA expression level seems to correlate with disease prognosis, and lower SPOP mRNA expression level showed a much worse prognosis, indicating the importance of SPOP expression level in PCa disease progression. In the next step, we will focus on the function and mechanism of SPOP in PCa.

With the rapid development of molecular biology research, there have been many diagnostic markers to help urologists detect PCa at the early stage. The most important marker is the prostate-specific antigen (PSA), which is a serine protease, also known as human kallikrein 3. PSA was first isolated and purified in 1979 and was introduced to clinical practice in 1986 [32]. PSA enters the blood and urine through the prostate catheter. Serum PSA could increase in some cases, such as urinary retention, prostate infection, benign prostatic hyperplasia and PCa. The role of serum PSA in the diagnosis of PCa has some limitations. To overcome these limitations, a few new molecular markers were also developed, such as prostatic acid phosphatase [33,34,35,36], miRNAs (such as PCA let-7 family) [37], transforming growth factor-β1 (TGF-β1) [38], fatty acid synthase [39,40] and PCA3 [41]. Moreover, a biomarker detection system composed of GalNaC-T3, PSMA, Hepsin and PCA3 could be a novel method to diagnose PCa [42,43]. The role of SPOP expression or SPOP mutation in PCa diagnosis is still unclear. In this study, we divided patients with PCa into SPOP mutant and SPOP wild-type groups and compared the clinical characteristics and prognosis between the two groups. The role of SPOP mutation in the diagnosis of PCa is an intriguing issue, which we will investigate in our future study.

Multiple pathways have been shown to be implicated in SPOP mutation. Many of them are cancer-related pathways, such as mTORC1 signaling pathway, p53 signaling pathway, NOTCH signaling, ROS pathway and KRAS pathway, indicating the potential role of SPOP mutation in disease progression. Previous study has shown the relationship between SPOP mutation and the mTOR signaling pathway [44]. The function of SPOP mutation in other cancer-related pathways is still unclear, and further studies are needed to investigate its mechanism.

In conclusion, this study identified the main pathways and genes associated with SPOP mutation in PCa, which may facilitate the development of SPOP mutation for expanding therapeutic strategies against PCa in men.
